# Closed-Loop Therapy and Sleep in Young People Newly Diagnosed With T1D and Their Parents

**DOI:** 10.1177/19322968241286816

**Published:** 2024-10-14

**Authors:** Juan J. Madrid-Valero, Eleanor M. Scott, Charlotte K. Boughton, Janet M. Allen, Julia Ware, Malgorzata E. Wilinska, Sara Hartnell, Ajay Thankamony, Tabitha Randell, Atrayee Ghatak, Rachel E.J. Besser, Daniela Elleri, Nicola Trevelyan, Fiona M. Campbell, Roman Hovorka, Alice M. Gregory

**Affiliations:** 1 Department of Human Anatomy and Psychobiology, University of Murcia, Murcia, Spain; 2 Biomedical Research Institute of Murcia, IMIB-Arrixaca, Murcia, Spain; 3 Leeds Institute of Cardiovascular and Metabolic Medicine, University of Leeds, Leeds, UK; 4 Metabolic Research Laboratories, Wellcome-MRC Institute of Metabolic Science, University of Cambridge, Cambridge, UK; 5 Wolfson Diabetes and Endocrine Clinic, Cambridge University Hospitals NHS Foundation Trust, Cambridge, UK; 6 Department of Paediatrics, University of Cambridge, Cambridge, UK; 7 Department of Paediatric Diabetes and Endocrinology, Nottingham Children’s Hospital, Nottingham, UK; 8 Department of Diabetes, Alder Hey Children’s NHS Foundation Trust, Liverpool, UK; 9 Department of Paediatrics, University of Oxford, Oxford, UK; 10 NIHR Oxford Biomedical Research Centre, John Radcliffe Hospital, Oxford, UK; 11 Department of Diabetes, Royal Hospital for Sick Children, Edinburgh, UK; 12 Paediatric Diabetes, Southampton Children’s Hospital, Southampton, UK; 13 Department of Paediatric Diabetes, Leeds Children’s Hospital, Leeds, UK; 14 Department of Psychology, Royal Holloway, University of London, Egham, UK

**Keywords:** actigraphy, closed loop, diabetes, diagnosis, sleep

## Abstract

**Background:**

A diagnosis of type 1 diabetes in a young person can create vulnerability for sleep. Historically it has been rare for young people to be offered a closed-loop system soon after diagnosis meaning that studies examining sleep under these circumstances in comparison with standard treatment have not been possible. In this study, we examine sleep in young people (and their parents) who were provided with hybrid closed-loop therapy at diagnosis of type 1 diabetes versus those who receive standard treatment over a 2-year period.

**Methods:**

The sample comprised 97 participants (mean age = 12.0 years; SD = 1.7) from a multicenter, open-label, randomized, parallel trial, where young people were randomized to either hybrid closed-loop insulin delivery or standard care at diagnosis. Sleep was measured using actigraphy and the Pittsburgh Sleep Quality Index (PSQI) in the young people, and using the PSQI in parents.

**Results:**

Sleep in young people using hybrid closed-loop insulin delivery did not differ significantly compared with those receiving standard care (although there were nonsignificant trends for better sleep in the closed-loop group for 4 of the 5 sleep actigraphy measures and PSQI). Similarly, there were nonsignificant differences for sleep between the groups at 24 months (with mixed direction of effects).

**Conclusions:**

This study assessed for the first time sleep in young people using a closed-loop system soon after diagnosis. Although sleep was not significantly different for young people using closed-loop insulin delivery as compared with those receiving standard care, the direction of effects of the nonsignificant results indicates a possible tendency for better sleep quality in the hybrid closed-loop insulin delivery group at the beginning of the treatment.

## Introduction

A diagnosis of type 1 diabetes (T1D) in a young person can create vulnerability for sleep within the family.^1^ Indeed, anxiety and life changes associated with this new diagnosis can cut into restful sleep.^2^ Even once initial challenges associated with the diagnosis have subsided, sleep may continue to be impacted over time because of requirements for night-time interventions to optimize blood glucose levels and other related factors. Type 1 diabetes may create sleep challenges for the young person living with the condition and can also affect caregivers such as parents.^1^ Indeed, parents can be centrally involved in the diabetes care of children, although adolescence is often a time of change for diabetes management, with young people beginning to take more responsibility for their condition.^3^ Short-term and long-term sleep difficulties for young people and their parents are noteworthy, as missing out on sleep and experiencing sleep of poor quality can have consequences for many different areas of life. Indeed, sleep is important for emotional regulation^4^ as well as memory and cognition.^5^ What is more, suboptimal sleep can have negative effects on various aspects of health including those directly relevant to diabetes such as insulin resistance and glucose metabolism which consequently affects glucose control.^6^

Different methods of treatment for diabetes are likely to impact sleep in different ways. A series of controlled trials have not found differences between hybrid closed-loop systems and sensor-augmented pumps using objective methods to assess sleep.^7-9^ However, several studies have found improvements in subjective sleep quality.^7,9^ Although further research is needed to understand the associations between treatment method and sleep, there are hypotheses as to why we might find associations. For example, the use of a closed-loop system with the ability to automatically tackle both hypoglycemia and hyperglycemia can reduce burden (less manual intervention means less sleep displacement). Closed-loop systems can also provide feelings of safety for young people and their parents^10^—which is so key for sleep.^11^ However, it is also possible that the learning curve to properly use the device can temporarily disrupt sleep as can associated alarms.^8,9^ Another possibility is that wearing an insulin pump can be uncomfortable for some and therefore can be a disturbance for sleep. Previous works comparing sleep in those using closed-loop systems and other treatments have provided mixed results. For example, self-reported sleep quality was better in adolescents when using hybrid closed-loop therapy with the Medtronic 780G advanced hybrid closed loop as compared with low-glucose suspend.^12^ Furthermore, in a study using a sample of both children and adults, it was found that the use of hybrid closed loop with the first-generation MiniMed 670G system (Medtronic) was also associated with sleep quality improvements.^13^ However, another study found that there were no significant differences in actigraphy-assessed sleep variables (except a reduction of number of parental awakenings during the night) in children using hybrid closed loop with Tandem Control IQ as compared with sensor-augmented pump therapy.^7^ Similarly, there was no difference in objectively assessed sleep quality and quantity in adolescents using hybrid closed loop with the 670G as compared with before they were using this system.^8^ There have also been mixed results when focusing on parental sleep. In the aforementioned study, as with the adolescents, parents of those using the hybrid closed-loop 670G did not show differences for objective sleep quality after their children had started using the closed-loop pump.^8^ However, self-report data revealed an improvement in parental sleep quality after closed-loop initiation.

A previous study by our group found a mixed pattern of nonsignificant results when examining the sleep quality and quantity (using both actigraphy data and questionnaires) in caregivers of children living with T1D using closed-loop insulin delivery with CamAPS FX as compared with sensor-augmented pump therapy.^14^ However, when considering the direction of effects, there was some indication of better sleep quality in the primary caregiver of children using closed loop. Further data from a multicenter trial focusing on the Control IQ closed-loop system found that the use of this device was associated with improved sleep in parents (of children living with T1D) who previously met the criteria for inadequate sleep.^15^ Similarly, use of the Omnipod 5 hybrid closed-loop system was associated with caregiver better self-reported sleep quality in certain domains.^16^ Furthermore, in an aforementioned study focusing on children using hybrid closed-loop with Control IQ as compared with sensor-augmented pump therapy,^7^ the closed-loop system was associated with better self-reported parental sleep (although there were not differences for actigraphy with the exception of night wakings).^7^

As historically it has been rare for young people to be offered a closed-loop pump soon after diagnosis, studies comparing sleep under these circumstances in comparison with those receiving standard treatment have not been possible. However, early adoption of a closed-loop system could reduce the burden of diabetes management and may have significant implications for sleep. Given mixed results to date, the limited research available on this topic, and the absence of information concerning the early adoption of the closed-loop system on sleep, further research using different closed-loop systems, age groups, and measures of sleep in young people and their parents are needed as are well-powered samples to elucidate this association further.

For these reasons, in this study, we examine sleep in young people commenced on hybrid closed-loop therapy at diagnosis of T1D (ie, within 21 days) versus those who receive standard treatment (for study details, see Boughton et al^17^) over a 24 months period. We also examine the sleep of their parents. We look to see whether both sleep quality and length differ in those using a closed-loop system and their parents as compared with those on standard treatment in both the short term (at the 6 months assessment) and longer term (at the 24 months assessment). We measure sleep using actigraphy (for the young people living with T1D) and using self-report (for both the young people and their parents).

## Methods

### Sample

This study compared sleep quality and quantity in young people who joined the study when they were newly diagnosed with T1D and their parents. Data came from the CLOuD study, a multicenter, open-label, randomized, parallel trial, where young people were randomized to either hybrid closed-loop insulin delivery (CL) or standard care (SC) soon after diagnosis.^17^ All control group participants were commenced on multiple daily injections at diagnosis but were free to commence insulin pump therapy and/or use flash/continuous glucose monitoring at any time following randomization. Treatment adjustments were made by local diabetes clinical teams (not the research team) as clinically indicated, applying National Institute for Health and Care Excellence criteria regarding eligibility for insulin pump therapy and/or glucose monitoring use. Interested readers can obtain more information about the treatment of the control group over the 24-month period elsewhere.^17^ The trial protocol and glycemic outcomes are also available elsewhere.^17^ Participants were recruited from pediatric diabetes clinics in the United Kingdom (Cambridge, Edinburgh, Leeds, Liverpool, Nottingham, Oxford, and Southampton). The key inclusion criterion for this study was a diagnosis of T1D within the previous 21 days and participant age from 10.0 to 16.9 years (full inclusion and exclusion criteria are available elsewhere).^17^ The total sample comprised 97 participants who were randomly allocated to receive CL (N = 51) or SC (N = 46) therapy over a period of 24 months. See the work by Boughton et al^17^ for further information and details about the CamAPS FX hybrid CL system.

Approval was received from the Cambridge East Research Ethics Committee (16/EE/0286) and the Medicines and Healthcare Products Regulatory Agency. Safety aspects were overseen by an independent data and safety monitoring board. The trial was co-coordinated by the Cambridge Clinical Trials Unit.

### Measures

#### Actiwatch data

Actigraphy (Philips Respironics, Bend, Oregon, USA) was used to obtain objective sleep data. Participants wore this device on the nondominant wrist for 7 consecutive nights (concomitantly with sleep diaries) and the mean scores for key variables are reported in analyses. Data were scored using the Philips ActiWare software version 6.0.9. To improve the accuracy of the actigraphy-derived sleep variables, the demarcation of the major rest intervals (ie, bed times and getting-up times) was manually edited using the intervals manipulation tool in the ActiWare software. Manual adjustment was done with the help of (1) sleep diaries; (2) markers from event marker button, if used within the actigraphs; (3) sharp changes in light level, if a light sensor is integrated within the actigraph, indicating light turned on/off at the time of waking-up/getting to sleep; as well as (4) sharp changes in activity count from/to sedentary level that usually accompanies sleep/wake activity pattern. After manual editing, the Philips software algorithms were then used to score the data. Data were scored using a 15-second epoch with default settings provided by the manufacturer (10 minutes of inactivity for onset of sleep and an awake threshold of 40 counts [medium]) to obtain standard measures of sleep continuity (1. total sleep time; 2. time in bed; 3. sleep efficiency [percentage of time spent asleep while in bed]; 4. sleep onset latency [time that it takes to change from wakefulness to sleep]; 5. number of awakenings; and 6. wake after sleep onset [WASO; time in wakefulness after having initially fallen asleep and prior to final awakening]). None of our participants reported extreme (±6 hours) disparities between the data provided by the actigraph and sleep diary or provided less than 70% data. Actigraphy data were measured for those living with T1D (but not their parents) at 2 different time points: (1) at 6 months after diagnosis (±2 weeks) and (2) between 21 and 24 months after diagnosis. Per protocol, data were also collected at 12 months, but for the purpose of this study, we decided to focus on the first and last assessments (6 and 24 months) to reduce the number of statistical analyses. Nonetheless, on request from a reviewer, we now briefly summarize findings from the 12 months assessment). Data from actigraphy were available for 71 and 61 participants at the 6- and 24-month time points, respectively. A sleep diary to verify actigraphy data was used which included the following questions: (1) enter the weekday; (2) at what time did you go to bed last night?; (3) after settling down, how long did it take you to fall asleep?; (4) after falling asleep, about how many times did you wake up in the night?; (5) after falling asleep, for how long were you awake during the night in total?; (6) at what time did you finally wake up?; (7) at what time did you get up?; (8) how would you rate the quality of your sleep last night? (with 5 response options from very poor [1] to very good [5]); and (9) times you took off the actiwatch. Participants who reported actigraphy data for less than 5 nights were removed.

#### Pittsburgh Sleep Quality Index

The Pittsburgh Sleep Quality Index (PSQI) is a widely used questionnaire to assess self-reported sleep quality during the previous month.^18^ The PSQI comprises 7 subscales: (1) subjective sleep quality, (2) sleep latency, (3) sleep duration, (4) habitual sleep efficiency, (5) sleep disturbances, (6) use of sleeping medication, and (7) daytime dysfunction. Scores on these 7 subscales range from 0 to 3 and are used to build a global score (ranging from 0 to 21) where higher scores represent poorer self-reported sleep quality.^18^ Subjects with a score higher than 5 points can be classified as having poor sleep quality. This questionnaire shows good psychometrics, scores correlate highly with objective measures of sleep,^19,20^ and has been validated in adolescent samples.^21^

### Statistical Analysis

All the analyses were done in R v.4.2.2. by the first author (JJM-V).^22^ Descriptive analyses were performed for actiwatch and questionnaire data. To test for significant differences between CL and SC, a series of *T*-tests were done. Sensitivity analyses where outliers were excluded were also performed. Outliers were identified as a score ±1.5 times the interquartile range (see Supplementary Table 1). In addition, regression models for measures at 24 months were fitted based on reviewers’ comments on an early draft of this article (so therefore represent a deviation from the original statistical plan). These models include baseline measures, age, sex, and study group (ie, CL/SC) to check whether treatment has an impact on sleep variables over the period of 24 months while controlling for baseline sleep measures.

## Results

The sample was 56.6% male and the mean age was 12.0 years (SD = 1.7) at baseline (Table 1). There were no significant differences between the CL and the SC groups for gender (*P* = .77), age (*P* = .20), or hemoglobin A_1C_ (HbA_1c_) levels pretreatment (*P* = .82). The HbA_1c_ levels were not associated with sleep quality nor sleep duration (both *P* > .05; at baseline and at 24 months assessment). There were also no differences regarding demographic variables for those who reported data and those who did not (*P* > .05). This sample has been shown to be representative of the general population of youths with newly diagnosed T1D (see the work by Boughton et al,^17^ Supplementary Table 15). Information from the PSQI was available for 60 young people living with T1D and 49 parents at the first assessment (6 months) and for 75 participants and 75 parents at the second assessment (24 months).

**Table 1.** Demographic Variables by Group.

	CL	SC	Total
Mean age (SD)	12.2 (1.56) Range 10-16	11.8 (1.69) Range 10-16	12 (1.63) Range 10-16
% male	54%	60%	57%
Ethnicity	85% white 6% unknown/not reported 4% Asian 2% black/African American 2% more than one selected	76% white 10% unknown/not reported 7% Asian 5% black/African American 2% more than one selected	81% white 8% unknown/not reported 6% Asian 3% black/African American 2% more than one selected

Abbreviations: CL, closed-loop therapy; SC, standard care.

### Six-Month Measure

*T* tests comparing sleep measures (actigraphy and PSQI) between groups were nonsignificant for all variables at 6 months (Table 2). When examining the direction of effects, participants from the CL group (as compared with the SC) showed longer sleep duration (x– = 7.9 [SD: 0.9] vs x– = 7.8 hours [SD: 0.7]), higher sleep efficiency (x– = 84.0% [SD: 4.3] vs x– = 83.0% [SD: 7.1]), shorter sleep onset latency (x– = 29.9 [SD: 18.1] vs x– = 36.6 minutes [SD: 37.3]), and fewer awakenings (x–=47.7 [SD: 12.9] vs x– = 51.2 [SD: 11.6]). Participants from the CL group, however, also showed longer WASO as compared with the SC group (x– = 36.5 [SD: 10.8] vs x–=35.5 minutes [SD: 12.1]). Regarding self-reported sleep quality from the PSQI, participants from the CL group reported better sleep quality (x– = 4.8 [SD: 2.5] vs x– = 5.5 [SD: 2.5]). Parents from the CL group reported slightly poorer sleep quality as compared with the SC group (x– = 6.0 [SD: 3.3] vs x–=5.5 [SD: 2.7]) (Table 2). Similar results were found when outliers were removed (Supplementary Table 1).

**Table 2.** Descriptive Statistics and Group Comparison.

	Measure (N for CL group/N for SC group)	CL	SC	*P*-value	Cohen’s D
Actigraphy data	Sleep duration 6 months (N = 40/31)	7.9 (SD = 0.9) hours	7.8 (SD = 0.7) hours	0.89	0.03
	Sleep duration 24 months (N = 36/25)	7.1 (SD = 1.0) hours	7.5 (SD = 2.0) hours	0.37	0.26
	Sleep efficiency 6 months (N = 40/31)	84.0% (SD = 4.3)	83.0% (SD = 7.1)	0.50	0.17
	Sleep efficiency 24 months (N = 36/25)	79.0% (SD = 8.0)	79.9 % (SD = 7.5)	0.67	0.11
	Wake after sleep onset 6 months (N = 40/31)	36.5 (SD = 10.8) minutes	35.5 (SD = 12.1) minutes	0.73	0.08
	Wake after sleep onset 24 months (N = 36/25)	36.9 (SD = 17.3) minutes	31.9 (SD = 10.3) minutes	0.16	0.34
	Latency 6 months (N = 40/31)	29.9 (SD = 18.1) minutes	36.6 (SD = 37.3) minutes	0.37	0.23
	Latency 24 months (N = 36/25)	44.0 (SD = 29.4) minutes	50.4 (SD = 32.0) minutes	0.44	0.21
	Nº of awakenings 6 months (N = 40/31)	47.7( SD = 12.9)	51.2 (SD = 11.6)	0.25	0.27
	Nº of awakenings 24 months (N = 36/25)	41.7 (SD = 13.5)	44.9 (SD = 10.4)	0.30	0.26
PSQI	Participant PSQI 6 months (N = 30/30)	4.8 (SD = 2.5)	5.5 (SD = 2.5)	0.26	0.26
	Participant PSQI 24 months (N = 41/34)	4.5 (SD = 2.1)	5.3 (SD = 3.4)	0.26	0.27
	Parent PSQI 6 months (N = 27/22)	6.0 (SD = 3.3)	5.5 (SD = 2.7)	0.55	0.17
	Parent PSQI 24 months (N = 41/34)	6.4 (SD = 3.8)	7.5 (SD = 3.1)	0.18	0.31

Abbreviations: CL, closed-loop therapy; PSQI, Pittsburgh Sleep Quality Index; SC, standard care.

Higher scores for PSQI represent poorer sleep quality. All data come from actigraphy except where it is stated that it comes from the PSQI. All measures refer to the young people living with type 1 diabetes, except for the PSQI which was also available for parents. Measures are available at 6 and 24 months postdiagnosis.

### 24-Month Measure

Again, *T* tests examining the group differences for all the sleep variables (actigraphy and PSQI) at 24 months were nonsignificant. When comparing the nonsignificant direction of effects only, some of the effect sizes were of a moderate magnitude (eg, sleep quality for parents and children as well as WASO; Table 2). In terms of the direction of effects at 24 months postdiagnosis, participants from the CL group as compared with the SC group showed shorter sleep onset latency (x– = 44.0 [SD: 29.4] vs x– = 50.4 minutes [SD: 32.0]), fewer awakenings (x– = 41.7 [SD: 13.5] vs x– = 44.9 [SD: 10.4]), and higher levels of sleep quality (self-reported) (x– = 4.5 [SD: 2.1] vs x– = 5.3 [SD: 3.4]). On the contrary, participants from the CL group (as compared with the SC group) showed shorter sleep duration (x– = 7.1 [SD: 1.0] vs x– = 7.5 hours [SD: 2.0]), slightly lower sleep efficiency (xx– = 79.0% [SD: 8.0] vs x– = 79.9% [SD: 7.5]), and longer WASO (x– = 36.9 [SD: 17.3] vs x– = 31.9 minutes [SD: 10.3]) (Table 2). Parents from the CL group reported better sleep quality (x– = 6.4 [SD: 3.8] vs x– = 7.5 [SD: 3.1]). Similar results were found when outliers were removed (Supplementary Table 1).

Study group (ie, CL/SC) was not a significant predictor (*P* > .05) in any of the regression models (actiwatch data, participants’ PSQI, and parents’ PSQI), including the baseline sleep measure, age, sex, and study group (CL vs SC) for sleep measures at 24 months. Finally, based on reviewers’ comments (not included in our statistical protocol), we also analyzed measures from the 12 months assessment. Again, we did not find significant differences between groups (CL/SC) except for number of awakenings which was marginally significant (*P* = .03) but nonsignificant after correcting for multiple testing.

Because of the relatively small sample size, the discussion of results below focuses on the direction of results and effect sizes rather than statistical significance, although it is important to keep in mind that none of the analyses reached significance at *P* < .05 (see Table 2).

## Discussion

In this study, we did not find significant differences between CL/SC groups on objective or self-reported sleep variables 6 and 24 months after diagnosis of T1D. When direction of effects and effect sizes were considered, young people using hybrid closed-loop appeared to have better sleep at 6 months after diagnosis as compared with those receiving SC (this was the case for 5 of the 6 sleep measures considered [sleep duration, sleep efficiency, latency, number of awakenings, and participant’s PSQI], although effect sizes were either very small or small for all variables, and none approached significance). In contrast, no indication of an advantage was found for the parent’s self-report of sleep quality in the closed-loop group (as compared with SC) when assessed at 6 months. There were similarly no significant group differences for sleep at 24 months. When direction of effects and effect sizes for differences in sleep were considered at 24 months, there was a much more mixed pattern of results, with certain aspects of sleep appearing to be slightly better in the closed-loop group, and other aspects of sleep appearing to be better in the SC group. Parents reported slightly better sleep quality at 24 months in the closed-loop group (vs the standard treatment group). Furthermore, treatment group was not a significant predictor in the regression analyses for sleep outcomes at 24 months while controlling for measures at 6 months.

The overall null results match other reports on this topic considering sleep assessed objectively.^7,8^ Null results could be explained by possible advantages of closed-loop technology for sleep being offset by increased alarm frequency, disrupting sleep, for example.^9^ Our null results do, however, contrast with some studies of closed-loop therapy, which show an advantage for self-reported sleep quality in both young people living with T1D^12^ and their parents.^8^ Although none of our group differences were significant, if there is a small advantage to sleep for the closed group early on in the diagnosis, which does not extend over time, there could be multiple explanations for this. For example, the early adoption of closed loop may bring advantages for sleep due to (1) increased feelings of safety and (2) less manual work (to address out of range blood glucose). Perhaps, group differences do not extend over time because these factors become less significant over time. For example, for the SC group, feelings of safety could increase over time because of the repeat confirmation of safety provided by waking up every morning. This could reflect the normalcy bias,^23^ which refers to the belief that normality will continue—and hence threats (in this case, dangerous night-time blood glucose levels) are underestimated. Another explanation is that the SC group could reduce their manual input over time because of burnout.^24^ Such hypotheses are speculative and would need to be tested—and do not explain the general overall trend that sleep does not appear to improve over time in our sample. Note that unlike other studies, here participants cannot compare their previous sleep against a different treatment as they were assigned directly to 1 treatment and therefore have never experienced anything other than CL.

The results should be considered in light of strengths and weaknesses of this study. Strengths of this report include the multicenter, randomized, longitudinal design of this study.^17^ Furthermore, the family context was considered by focusing on sleep in both young people living with T1D and their parents. In addition, young people were enrolled shortly after diagnosis (although we acknowledge that sleep was measured for the first time at 6 months after diagnosis and was not assessed from the outset of the study). Finally, the developmental stage of those living with T1D represents an interesting time given that this period is associated with an increased shift from parental to self-care. A further strength of this report is that both self-report and actigraphy were used to assess sleep in young people. A limitation of this work is that we did not attempt to assess factors influencing sleep in different treatment groups (eg, reports of manual interventions to address diabetes; alarms). It is also noteworthy that parent sleep was only measured using self-report. We also acknowledge that while used as standard in studies, some actigraphy measures such as number of awakenings are highly sensitive to movement and may not represent true awakenings. A further limitation is seasonality which could impact sleep measures, this variable was not considered although we do not expect seasonality to have impacted the sleep in one group to a greater extent than another. Future work needs to consider such factors systemically to allow a comprehensive understanding of the night-time experiences of those living with T1D and their family members. A further consideration is that, while the sample size was appropriate to consider the main aims of the study (effect on preservation of C-peptide secretion with closed-loop therapy in young people newly diagnosed with T1D), it may be that if there is in fact an impact of closed-loop therapy on sleep it may be small, and hence larger sample sizes are needed to detect this. Even small changes in sleep over substantial time-periods have the potential to make meaningful differences to well-being—so need to be explored.

## Conclusions

In conclusion, this study provides novel results about sleep in newly diagnosed young people that have never experienced any treatment other than a CL system and those newly diagnosed who were given standard treatment. Although sleep was not significantly different for young people using closed-loop insulin delivery as compared with those receiving SC, there seems to be a tendency for better sleep quality in the CL group at 6 months after diagnosis and the beginning of the treatment. Careful consideration of the data suggests that further study using a larger sample, further measurements of sleep, longitudinal data, and an assessment of multiple factors impacting sleep could prove beneficial.

## Supplemental Material

sj-docx-1-dst-10.1177_19322968241286816 – Supplemental material for Closed-Loop Therapy and Sleep in Young People Newly Diagnosed With T1D and Their Parents Supplemental material, sj-docx-1-dst-10.1177_19322968241286816 for Closed-Loop Therapy and Sleep in Young People Newly Diagnosed With T1D and Their Parents by Juan J. Madrid-Valero, Eleanor M. Scott, Charlotte K. Boughton, Janet M. Allen, Julia Ware, Malgorzata E. Wilinska, Sara Hartnell, Ajay Thankamony, Tabitha Randell, Atrayee Ghatak, Rachel E.J. Besser, Daniela Elleri, Nicola Trevelyan, Fiona M. Campbell, Roman Hovorka and Alice M. Gregory in Journal of Diabetes Science and Technology
