# Supplementary material for: Closed-Loop Therapy and Sleep in Young People Newly Diagnosed With Type 1 Diabetes and Their Parents
Source: J Diabetes Sci Technol. 2024 Oct 14;20(2):335–41. doi: 10.1177/19322968241286816 (PMC11571613; doi:10.1177/19322968241286816)
Supplement: sj-docx-1-dst-10.1177_19322968241286816 – Supplemental material for Closed-Loop Therapy and Sleep in Young People Newly Diagnosed With T1D and Their Parents [file sj-docx-1-dst-10.1177_19322968241286816.docx]

Supplementary Table 1: descriptive statistics and group comparison (outliers removed)

|  | Measure (N for CL group/ N for SC group) | CL | SC | p-value | Cohen’s D |
| --- | --- | --- | --- | --- | --- |
| Actigraphy data | Sleep duration 6 months (N=40/30) | 7.8 (SD=0.9) hours | 7.9 (SD=0.6) hours | 0.82 | 0.05 |
|  | Sleep duration 24 months (N=33/23) | 7.3 (SD=0.73) hours | 7.2 (SD=0.6) hours | 0.61 | 0.13 |
|  | Sleep efficiency 6 months (N=40/29) | 84.0% (SD=4.3) | 84.3% (SD=5.1) | 0.79 | 0.07 |
|  | Sleep efficiency 24 months (N=35/24) | 79.9% (SD=6.4) | 80.8 % (SD=6.4) | 0.61 | 0.13 |
|  | Wake after sleep onset 6 months (N=40/30) | 36.5 (SD=10.8) mins | 34.4 (SD=10.7) mins | 0.43 | 0.19 |
|  | Wake after sleep onset 24 months (N=33/25) | 33.1 (SD=12.0) mins | 31.9 (SD=10.3) mins | 0.68 | 0.11 |
|  | Latency 6 months (N=40/30) | 29.9 (SD=18.1) mins | 30.8 (SD=19.7) mins | 0.85 | 0.05 |
|  | Latency 24 months (N=40/31) | 44.0 (SD=29.4) mins | 50.4 (SD=32.0) mins | 0.44 | 0.21 |
|  | Nº of awakenings 6 months (N=39/31) | 46.8(SD=11.6) | 51.2 (SD=11.6) | 0.12 | 0.37 |
|  | Nº of awakenings 24 months (N=36/25) | 41.7 (SD=13.5) | 45.0 (SD=10.4) | 0.30 | 0.26 |
| PSQI | Participant PSQI 6 months (N=30/30) | 4.8 (SD=2.5) | 5.5 (SD=2.5) | 0.26 | 0.29 |
|  | Participant PSQI 24 months (N=41/32) | 4.5 (SD=2.1) | 4.8 (SD=2.7) | 0.67 | 0.10 |
|  | Parent PSQI 6 months (N=26/22) | 5.6 (SD= 2.8) | 5.5 (SD= 2.7) | 0.84 | 0.06 |
|  | Parent PSQI 24 months (N=41/32) | 6.4 (SD= 3.8) | 7.5 (SD= 3.1) | 0.18 | 0.31 |

CL: Closed-loop therapy; SC: Standard Care; PSQI: Pittsburgh Sleep Quality Index.

*Higher scores for PSQI represent poorer sleep quality

*All data comes from actigraphy except where it is stated that it comes from the PSQI

*All measures refer to the young people living with type 1 diabetes, except for the PSQI which was also available for parents.

*Measures are available at 6 and 24 months post diagnosis.
